# Heterozygous connexin 50 mutation affects metabolic syndrome attributes in spontaneously hypertensive rat

**DOI:** 10.1186/s12944-016-0376-3

**Published:** 2016-11-21

**Authors:** Ondřej Šeda, Drahomíra Křenová, Olena Oliyarnyk, Lucie Šedová, Michaela Krupková, František Liška, Blanka Chylíková, Ludmila Kazdová, Vladimír Křen

**Affiliations:** 1 Institute of Biology and Medical Genetics, First Faculty of Medicine, Charles University, Albertov 4, 128 00 Prague 2, Czech Republic; 2 Division BIOCEV, Institute of Molecular Genetics of the Academy of Sciences of the Czech Republic, Laboratory of Rat Models of Metabolic Disorders, Vídeňská 1083, 142 20 Prague 4, Czech Republic; 3 Center for Experimental Medicine, Institute for Clinical and Experimental Medicine, Vídeňská 1958/9, 140 21 Prague 4, Czech Republic

**Keywords:** Connexin, Animal models, Lipoprotein, Oxidative stress, Metabolic syndrome

## Abstract

**Background:**

Several members of connexin family of transmembrane proteins were previously implicated in distinct metabolic conditions. In this study we aimed to determine the effects of complete and heterozygous form of connexin50 gene (*Gja8*) mutation L7Q on metabolic profile and oxidative stress parameters in spontaneously hypertensive inbred rat strain (SHR).

**Methods:**

Adult, standard chow-fed male rats of SHR, heterozygous SHR-*Dca*+/− and SHR-*Dca*−/− coisogenic strains were used. At the age of 4 months, dexamethasone (2.6 μg/ml) was administered in the drinking water for three days. The lipidemic profile (cholesterol and triacylglycerol concentration in 20 lipoprotein fractions, chylomicron, VLDL, LDL and HDL particle sizes) together with 33 cytokines and hormones in serum and several oxidative stress parameters in plasma, liver, kidney and heart were assessed.

**Results:**

SHR and SHR-*Dca*−/− rats had similar concentrations of triacylglycerols and cholesterol in all major lipoprotein fractions. The heterozygotes reached significantly highest levels of total (SHR-*Dca*+/−: 51.3 ± 7.2 vs. SHR: 34.5 ± 2.4 and SHR-*Dca*−/−: 34.4 ± 2.5 mg/dl, *p* = 0.026), chylomicron and VLDL triacylglycerols. The heterozygotes showed significantly lowest values of HDL cholesterol (40.9 ± 2.3 mg/dl) compared both to SHR (51.8 ± 2.2 mg/dl) and SHR-*Dca*−/− (48.6 ± 2.7 mg/dl). Total and LDL cholesterol in SHR-*Dca*+/− was lower compared to SHR. Glucose tolerance was improved and insulin concentrations were lowest in SHR-*Dca*−/− (1.11 ± 0.20 pg/ml) in comparison with both SHR (2.32 ± 0.49 pg/ml) and SHR-*Dca*+/− (3.04 ± 0.21 pg/ml). The heterozygous rats showed profile suggestive of increased oxidative stress as well as highest serum concentrations of several pro-inflammatory cytokines including interleukins 6, 12, 17, 18 and tumor necrosis factor alpha.

**Conclusions:**

Our results demonstrate that connexin50 mutation in heterozygous state affects significantly the lipid profile and the oxidative stress parameters in the spontaneously hypertensive rat strain.

**Electronic supplementary material:**

The online version of this article (doi:10.1186/s12944-016-0376-3) contains supplementary material, which is available to authorized users.

## Background

The rapidly increasing incidence and prevalence of dyslipidemia, obesity and type 2 diabetes is becoming a major global health issue, however, the need for identification of relevant molecular targets and devising effective preventive and therapeutic algorithms is met by only modest advancement of our knowledge. Historically, several major definitions of metabolic syndrome have been used, yet in 2009, five major scientific organizations released a joint interim statement leading to a unified definition of the disorder [1]. Individuals who meet at least 3 out of 5 criteria [elevated waist circumference, elevated triglycerides, reduced high-density lipoprotein cholesterol, elevated blood pressure and elevated fasting glucose (or treatment of these conditions)] are diagnosed with metabolic syndrome. Exact thresholds are defined for all criteria, except elevated waist circumference, which must rely on population and country-specific definitions [1]. All individual features of metabolic syndrome are complex traits with relatively balanced strengths of heritable and environmental components (the heritability of individual components ranges from 40–70%) [2]. The detailed analysis of genetic component of complex traits in general human population encounters numerous hurdles, including reduced penetrance, expressivity, ethnical admixture and other population stratifications, epigenetic and environmental influences [3]. Also, it is not only the fully manifested condition that is of clinical relevance; evidence is mounting with regard to the importance of early stages of metabolic syndrome components, particularly impaired glucose tolerance and prediabetes [4]. In this context, experimental models can greatly facilitate the process of deconstruction of genetic architecture of multifactorial traits [5]. In rodents and humans, a family of highly conserved transmembrane proteins forming intercellular gap junction channels called connexins comprises 21 isoforms [6]. The tissue distribution pattern of many connexins and their close functional connection to multiple metabolic and signaling pathways that are crucial for insulin sensitivity, lipid homeostasis and hemodynamic regulation makes them plausible candidates for metabolic syndrome pathogenesis [7]. We have previously established that a spontaneous mutation L7Q in *Gja8* gene (coding for connexin50) which arose in spontaneously hypertensive rat strain (SHR hereafter; Rat Genome Database (RGD) [8] ID: 631848) leads to microphthalmia and cataract [9]. The resulting mutant coisogenic strain SHR-Gja8^m1Cub^ (SHR-*Dca*−/− hereafter, RGD ID: 2293729) shows decreased blood pressure compared to SHR [10]. This study aimed to further explore the effects of connexin50 mutation on metabolic and cytokine profile in SHR-*Dca*−/− and SHR-*Dca*+/− strains including a battery of parameters of oxidative stress in the animals challenged by dexamethasone, a dyslipidemia and insulin resistance-inducing glucocorticoid [11, 12].

## Methods

### Experimental protocol

Adult male rats were housed under temperature- and humidity-controlled conditions with a 12 h/12 h light–dark cycle. Animals had free access to food (standard chow) and water at all times. At 4 months of age, males from the SHR, SHR-*Dca*+/− and SHR-*Dca*−/− strains (*n* = 8/strain/procedure) were administered dexamethasone (Dexamed, Medochemie) in drinking water (2.6 μg/ml) for three days as described previously [11, 12]. Subsequently, they were subjected to an oral glucose tolerance test (OGTT) after overnight fasting and blood samples were drawn. The animals were then sacrificed, and their total weight and the weights of the heart, liver, kidneys, and adrenal glands, and the epididymal and retroperitoneal fat pads, were determined.

### Metabolic measurements

The OGTT was performed after overnight fasting. Blood samples for glycemic assessment (Ascensia Elite Blood Glucose Meter, Bayer HealthCare, Mishawaka, IN, USA; validated by the Institute of Clinical Biochemistry and Laboratory Diagnostics of the First Faculty of Medicine, Charles University in Prague) were obtained from the tail vein at intervals of 0, 30, 60, 120, and 180 min after intragastric glucose administration to conscious rats (3 g/kg body weight, 30% aqueous solution). The lipid profile (cholesterol and triacylglycerols blood concentration in 20 lipoprotein fractions, glycerol level and chylomicron, VLDL, LDL and HDL particle sizes) was assessed by high performance liquid chromatography method as described previously [13, 14]. Enzyme-linked immunosorbent assay (ELISA) kits were used to determine the serum levels of adiponectin (BioSource, San Diego, CA, USA). Milliplex Rat Metabolic Hormone Magnetic Bead panel (Merck Millipore, Darmstadt, Germany) was used for the simultaneous quantification of C-peptide, gastric inhibitory polypeptide (GIP), glucagon-like polypeptide-1 (GLP1), pancreatic polypeptide (PP), protein tyrosine tyrosine (PYY), glucagon, insulin and leptin, the Bio-Plex Pro Rat Cytokine 24-Plex Immunoassay (Bio-Rad, Hercules, CA, USA) was used to assess the concentrations of erythropoietin (EPO), Granulocyte-colony stimulating factor (G-CSF), Granulocyte-macrophage colony-stimulating factor (GM-CSF), chemokine (C-X-C motif) ligand 1 (GRO/KC), interferon gamma (IFN-γ), interleukins IL-1α, IL-1β, IL-2, IL-4, IL-5, IL-6, IL-7, IL-10, IL-12p70, IL-13, IL-17A, IL-18, macrophage colony-stimulating factor (M-CSF), monocyte chemotactic protein 1 (MCP-1), macrophage inflammatory protein 1-alpha (MIP-1α), macrophage inflammatory protein 3-alpha (MIP-3α), regulated on activation, normal T cell expressed and secreted (RANTES), tumor necrosis factor alpha (TNF-α) and vascular endothelial growth factor (VEGF), using BioPlex system (Bio-Rad, Hercules, CA, USA).

The oxidation stress parameters were assessed as described previously [15, 16]. In short, the activity of superoxide dismutase (SOD) was determined spectrophotometrically using p-tetrazolin nitro-blue, the activity of catalase (CAT) was measured spectrophotometrically by ammonium molybdate reaction with H_2_O_2_, and the activity of glutathione peroxidase (GSH-Px) and content of glutathione (GSH) were analyzed spectrophotometrically using Ellman’s reagent. Glutathione reductase activity was measured using commercially available assay kit (Sigma-Aldrich, St. Louis, MO, USA). The level of conjugated dienes (CD) was determined spectrophotometrically after extraction into n-heptane. Thiobarbituric acid reactive substances (TBARS) were measured according to Naito et al. [17].

### Statistical analysis

All statistical analyses were performed using STATISTICA 12 CZ. The metabolic and morphometric data were compared by one-way analyses of variance (ANOVA) with STRAIN as main factor followed by Tukey’s honest significance difference test for detailed pair-wise comparison. The null hypothesis was rejected whenever *p* < 0.05.

## Results

### Morphometric and metabolic profile

**Fig. 1:**
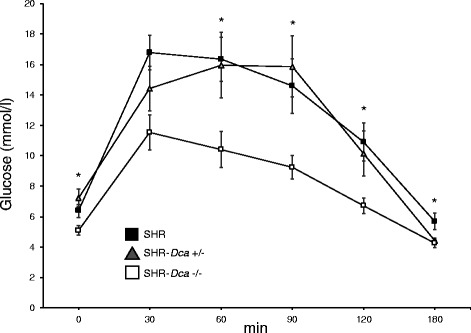
Glycemic time courses for adult male SHR (black squares) vs. SHR-*Dca*+/− (grey triangles) and SHR-*Dca*−/− (white squares) adult male rats. Data are expressed as mean ± SEM (*n* = 8/strain). Within the graph, the significance levels of the ANOVA for STRAIN as major factor are indicated as follows: * *p* < 0.05

The body weights and the relative weights of major organs were comparable among the three genotypes. The relative weight of the adipose tissue depots was decreased in the SHR-*Dca*−/− rats and increased in the heterozygotes compared to SHR, in both cases reaching statistical significance for the epididymal fat pad (Table 1). Glucose tolerance was improved in SHR-*Dca*−/− in comparison to both SHR and SHR-*Dca*+/− strains as reflected by significantly lowest values of glycaemia throughout the oral glucose tolerance test (Fig. 1) combined with lower insulin and C-peptide concentrations (Table 1). The heterozygous rats showed the highest concentrations of gastric inhibitory polypeptide of the three tested strains. Glucagon, glucagon-like polypeptide-1, pancreatic polypeptide, protein tyrosine tyrosine, adiponectin and leptin levels did not differ among the three strains (Table 1).

**Table 1:** Morphometric and metabolic profile of SHR, SHR-*Dca* +/− and SHR-*Dca* −/− male rats

Trait	SHR	SHR-*Dca*+/−	SHR-*Dca*−/−	*p*-value
Body weight (b.wt.), g	299 ± 6	299 ± 9	281 ± 8	0.16
Liver, g/100 g b.wt.	3.19 ± 0.02	3.29 ± 0.08	3.16 ± 0.09	0.41
Heart, g/100 g b.wt.	0.44 ± 0.01	0.45 ± 0.01	0.45 ± 0.01	0.99
Kidney, g/100 g b.wt.	0.77 ± 0.01	0.77 ± 0.01	0.77 ± 0.01	0.98
Adrenals, mg/100 g b.wt.	8.46 ± 0.04	8.50 ± 0.04	9.58 ± 0.07	0.21
EFP wt., g/100 g b.wt.	1.05 ± 0.03	1.14 ± 0.03 ^*b*^	0.92 ± 0.05*	*0.005*
RFP wt., g/100 g b.wt.	0.91 ± 0.05	0.98 ± 0.05	0.79 ± 0.09	0.16
Glycerol (mg/dl)	2.97 ± 0.32	2.73 ± 0.23	3.12 ± 0.30	0.67
Adiponectin (μg/ml)	5.14 ± 0.56	5.58 ± 0.30	5.42 ± 0.24	0.72
C-peptide, pg/ml	615 ± 57	653 ± 39^*a*^	425 ± 42*	*0.046*
GIP, pg/ml	163 ± 25	247 ± 33*^,*a*^	122 ± 20	*0.031*
GLP1, pg/ml	102 ± 56	68 ± 41	71 ± 44	0.86
Glucagon, pg/ml	155 ± 50	365 ± 111	153 ± 35	0.10
Insulin, ng/ml	2.32 ± 0.49	3.04 ± 0.21^*b*^	1.11 ± 0.20*	*0.025*
PP, pg/ml	39.3 ± 3.9	45.1 ± 4.3	39.2 ± 3.5	0.52
PYY, pg/ml	79.3 ± 10.0	96.3 ± 15.6	76.6 ± 13.5	0.53
Leptin, ng/ml	6.38 ± 0.39	6.51 ± 0.35	6.41 ± 0.52	0.97

Data are shown as mean ± SEM. The significance levels of one-way ANOVA for STRAIN as a major factor are shown in the last column; significant values are italicized. * *p* < 0.05 for pair-wise comparisons (post-hoc Tukey’s HSD test) between SHR-*Dca*−/− or SHR-*Dca*+/− strains vs. SHR. ^*a*^ *p* < 0.05 and ^*b*^ *p* < 0.01 for pair-wise comparisons between SHR-*Dca*−/− and SHR-*Dca*+/− strains. b.wt: body weight; *EFP* epididymal fat pad, *RFP* retroperitoneal fat pad, *GIP* gastric inhibitory polypeptide, *GLP1* glucagon-like polypeptide-1, *PP* pancreatic polypeptide, *PYY* protein tyrosine tyrosine. * *p < 0.05 vs. SHR.* ^*a*^ *p* < *0.05 vs. SHR-Dca−/−.* ^*b*^ *p* < *0.01 vs. SHR-Dca−/−*

### Detailed lipid profile

**Fig. 2:**
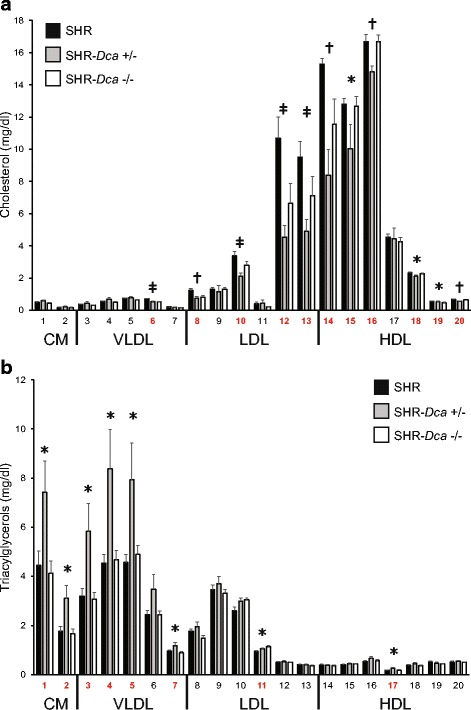
The cholesterol (panel **a**) and triacylglycerols (panel **b**) content in 20 lipoprotein subfractions in SHR (black bars), SHR-*Dca*+/− (grey bars) and SHR-*Dca*−/− (open bars) adult male rats. Data are expressed as mean ± SEM (*n* = 8/strain). Within the graph, the significance levels of the ANOVA for STRAIN as major factor are indicated as follows: * *p* < 0.05; **†** *p* < 0.01; **‡** *p* < 0.001. Labels of fractions showing significant differences between strains are highlighted in red. The allocation of individual lipoprotein subfractions to major lipoprotein classes is shown in order of particle’s decreasing size from left to right. CM: chylomicron, VLDL: very low-density lipoprotein, LDL: low-density lipoprotein, HDL: high-density lipoprotein

While SHR and SHR-*Dca*−/− rats had similar concentrations of triacylglycerols and cholesterol in all major lipoprotein fractions, the heterozygotes reached significantly highest levels of total, chylomicron (CM) and very low-density lipoprotein (VLDL) triacylglycerols of the three tested strains (Table 2). As shown in Fig. 2, this difference was evident throughout most CM and VLDL subfractions, further corroborated by significantly largest VLDL particles in SHR-*Dca*+/− (Additional file 1: Table S1). On the other hand, total and low-density lipoprotein (LDL) cholesterol in SHR-*Dca*+/− was lower compared to SHR and for high-density lipoprotein (HDL) cholesterol the heterozygotes showed significantly lowest values compared both to SHR and SHR-*Dca*−/− (Table 2). The detailed analysis revealed that most pronounced dissimilarities for cholesterol concentrations were found in small and very small LDL particles and in very large HDL particles (Fig. 2).

**Table 2:** Triacylglycerols and cholesterol concentrations in major lipoprotein subfractions in SHR, SHR-*Dca*+/− and SHR-*Dca*−/− male rats

Trait (mg/dl)	SHR	SHR-*Dca*+/−	SHR-*Dca*−/−	*p*-value
Total TG	34.5 ± 2.4	51.3 ± 7.2*^, *a*^	34.4 ± 2.5	*0.026*
Chylomicron TG	6.20 ± 0.87	10.53 ± 1.80*^, *a*^	5.78 ± 0.79	*0.030*
VLDL-TG	15.7 ± 1.3	26.8 ± 4.9*^, *a*^	16.0 ± 1.4	*0.025*
LDL-TG	9.63 ± 0.55	9.98 ± 0.76	9.84 ± 0.32	0.91
HDL-TG	2.89 ± 0.22	3.19 ± 0.21	2.84 ± 0.13	0.42
Total C	81.4 ± 5.2	58.0 ± 3.2†	70.2 ± 5.7	*0.016*
Chylomicron C	0.70 ± 0.07	0.83 ± 0.09	0.63 ± 0.06	0.19
VLDL-C	2.64 ± 0.12	2.67 ± 0.30	2.19 ± 0.15	0.22
LDL-C	26.2 ± 3.0	13.6 ± 1.3†	18.7 ± 3.0	*0.015*
HDL-C	51.8 ± 2.2	40.9 ± 2.3†^, *a*^	48.6 ± 2.7	*0.020*

Triacylglycerols (TG) and cholesterol (C) concentrations in major lipoprotein subfractions (chylomicron, *VLDL* very low-density lipoprotein, *LDL* low-density lipoprotein, *HDL* high-density lipoprotein) in SHR, SHR-Dca+/− and SHR-Dca−/− male rats. Data are shown as mean ± SEM. The significance levels of one-way ANOVA for STRAIN as a major factor are shown in last column; significant values are italicized. * *p* < 0.05 and † *p* < 0.01 for pair-wise comparisons (post-hoc Tukey’s HSD test) between SHR-*Dca*−/− or SHR-*Dca*+/− strains vs. SHR. ^*a*^ *p* < 0.05 for pair-wise comparisons between SHR-*Dca*−/− and SHR-*Dca*+/− strains. * *p < 0.05 vs. SHR.* † *p* < *0.01 vs. SHR.* ^*a*^ *p* < *0.05 vs. SHR-Dca−/−*

### Oxidative stress assessment and cytokine profile

**Fig. 3:**
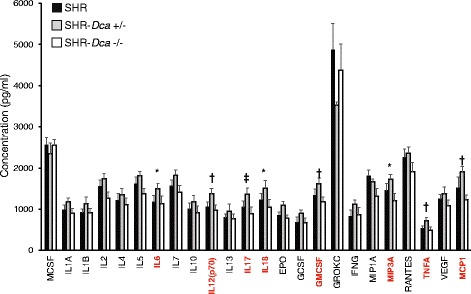
The cytokine concentrations in SHR (black bars) vs. SHR-*Dca*+/− (grey bars) and SHR-*Dca*−/− (open bars) adult male rats. Data are expressed as mean ± SEM (*n* = 8/strain). Within the graph, the significance levels of the ANOVA for STRAIN as major factor are indicated as follows: * *p* < 0.05; **†** *p* < 0.01; **‡** *p* < 0.001. Labels of traits showing significant differences between strains are highlighted in red EPO: erythropoietin, G-CSF: granulocyte-colony stimulating factor, GM-CSF: granulocyte-macrophage colony-stimulating factor, GRO/KC: chemokine (C-X-C motif) ligand 1, IFN-γ: interferon gamma, IL: interleukins, M-CSF: macrophage colony-stimulating factor, MCP-1: monocyte chemotactic protein 1, MIP-1α: macrophage inflammatory protein 1-alpha, MIP-3α: macrophage inflammatory protein 3-alpha, RANTES: regulated on activation, normal T cell expressed and secreted, TNF-α: tumor necrosis factor alpha, VEGF: vascular endothelial growth factor

SOD activity in liver and plasma GSH were significantly decreased both in SHR-*Dca*+/− and SHR-*Dca*−/− compared to SHR (Table 3). TBARS in plasma were significantly increased both in SHR-*Dca*+/− and SHR-*Dca*−/− compared to SHR. While the SHR-*Dca*−/− showed comparable values to those in SHR for the rest of the assessed oxidative stress parameters, the heterozygous rats had significantly lowest activity of kidney SOD, kidney glutathione peroxidase and plasma glutathione reductase as well as significantly highest liver catalase activity compared both to SHR and SHR-*Dca*−/− (Table 3). Also, plasma glutathione peroxidase activity was lower in SHR-*Dca*+/− compared to SHR. While SHR and SHR-*Dca*−/− did not differ across the panel of 24 cytokines, the SHR-*Dca*+/− showed significantly highest concentrations of interleukins IL6, IL12, IL17, IL18, granulocyte-macrophage colony-stimulating factor, macrophage inflammatory protein 3-alpha and monocyte chemotactic protein 1 (Fig. 3).

**Table 3:** Oxidative stress parameters in SHR, SHR-*Dca*+/− and SHR-*Dca*−/− male rats

Phenotype	SHR	SHR-Dca+/−	SHR-Dca−/−	*p*-value
Plasma SOD	0.343 ± 0.030	0.319 ± 0.030	0.283 ± 0.034	0.43
Liver SOD	0.097 ± 0.006	0.074 ± 0.005†	0.082 ± 0.003*	*0.012*
Kidney SOD	0.070 ± 0.002	0.040 ± 0.002‡^,*b*^	0.059 ± 0.008	*0.0010*
Heart SOD	0.085 ± 0.008	0.079 ± 0.009	0.083 ± 0.009	0.88
Plasma CAT	341 ± 16	354 ± 30	414 ± 55	0.36
Liver CAT	773 ± 37	1081 ± 24‡^,*c*^	827 ± 31	*0.000004*
Kidney CAT	464 ± 19	571 ± 41	539 ± 46	0.16
Heart CAT	629 ± 36	679 ± 31	621 ± 38	0.44
Plasma GSH-Px	546 ± 30	379 ± 27†	426 ± 60	*0.027*
Liver GSH-Px	417 ± 27	320 ± 18*	389 ± 33	*0.042*
Kidney GSH-Px	506 ± 31	378 ± 23†^,*a*^	460 ± 24	*0.0092*
Heart GSH-Px	624 ± 35	577 ± 20	612 ± 16	0.38
Plasma GR	212 ± 21	158 ± 13*^,*c*^	259 ± 13	*0.001*
Liver GR	387 ± 22	430 ± 14	372 ± 32	0.20
Kidney GR	273 ± 16	241 ± 9	198 ± 31	0.06
Heart GR	244 ± 24	228 ± 16	192 ± 26	0.27
Plasma GSH	31.0 ± 2.0	22.3 ± 1.1‡	21.9 ± 1.0‡	*0.0004*
Liver GSH	24.6 ± 1.5	23.4 ± 1.3	28.2 ± 1.8	0.10
Kidney GSH	25.1 ± 1.7	19.8 ± 1.2	24.6 ± 1.9	0.06
Heart GSH	25.8 ± 2.4	27.4 ± 2.0	25.5 ± 1.2	0.75
Plasma TBARS	1.72 ± 0.05	2.32 ± 0.13†	2.24 ± 0.13†	*0.0030*
Liver TBARS	0.62 ± 0.04	1.05 ± 0.09‡	0.87 ± 0.05*	*0.0016*
Kidney TBARS	1.27 ± 0.11	1.49 ± 0.14	1.54 ± 0.16	0.37
Heart TBARS	1.88 ± 0.11	2.38 ± 0.23	1.97 ± 0.23	0.20
Plasma CD	31.6 ± 2.7	34.8 ± 1.5	33.0 ± 2.0	0.54
Liver CD	33.7 ± 1.0	32.1 ± 1.6	29.1 ± 2.2	0.18
Kidney CD	19.8 ± 2.3	20.7 ± 1.6	20.8 ± 2.3	0.93
Heart CD	15.1 ± 1.1	19.5 ± 1.4	19.1 ± 1.9	0.11

The parameters of oxidative stress in plasma, liver, kidney cortex and heart of SHR, SHR-*Dca*+/− and SHR-*Dca*−/− adult male rats are shown as mean ± SEM. The significance levels of one-way ANOVA for STRAIN as a major factor are shown in the last column; significant values are italicized. * *p* < 0.05, † *p* < 0.01 and ‡ *p* < 0.001 for pair-wise comparisons (post-hoc Tukey’s HSD test) between SHR-*Dca*−/− or SHR-*Dca*+/− strains vs. SHR. ^*a*^ *p* < 0.05, ^*b*^ *p* < 0.01 and ^*c*^ *p* < 0.001 for pair-wise comparisons between SHR-*Dca*−/− and SHR-*Dca*+/− strains. Superoxide dismutase (SOD) units : U I ml^−1^ (plasma), U I mg prot^−1^ (liver, kidney, heart); Catalase (CAT) units: μM H_2_O_2_ min^−1^ ml^−1^ (plasma), μM H_2_O_2_ min^−1^mg prot^−1^ (liver, kidney, heart); Glutathione peroxidase (GSH-Px) units μM GSH min^−1^ml^−1^ (plasma), μM GSH min^−1^mg prot^−1^ (liver, kidney, heart); Glutathione reductase (GR) units: nM NADPH min^−1^ml^−1^ (plasma), nM NADPH min^−1^mg prot^−1^ (liver, kidney, heart); Glutathione (GSH) units: nM ml^−1^ (plasma), μM mg prot^−1^ (liver, kidney, heart); Thiobarbituric acid reactive substances (TBARS) units nM ml^−1^ (plasma), nM mg prot^−1^ (liver, kidney, heart); conjugated dienes (CD) units: nM ml^−1^ (plasma), nM mg prot^−1^ (liver, kidney, heart). * *p < 0.05 vs. SHR.* ^*a*^ *p* < *0.05 vs. SHR-Dca−/−.* † *p* < *0.01 vs. SHR.* ^*b*^ *p* < *0.01 vs. SHR-Dca−/−.* ‡ *p* < *0.001 vs. SHR.* ^*c*^ *p* < *0.001 vs. SHR-Dca−/−*

## Discussion

The results of the current study demonstrate that the nonsynonymous mutation L7Q in the amino terminal domain of Cx50 has multiple metabolic consequences in spontaneously hypertensive rat and these vary substantially depending on the homozygous vs. heterozygous state of the mutation. The distinct phenotypic effect of heterozygous genotype has been previously observed for another member of connexin family, the *GJA5* gene coding for connexin 40. While several heterozygous *GJA5* mutations were identified in patients suffering from atrial fibrillation, transgenic mice carrying one of these mutations, Cx40A96S, developed hypertension only when both alleles were mutant [18]. Further studies suggest that the gene dosage effect may be also tissue-specific [19]. It is presumed that mutations in the regions contributing to channel pore are likely to result in functional alterations [20]. In a comprehensive review of available structural and functional studies of Cx50 gap junction channels, Xin and Bai summarized compelling evidence that the amino terminal domain of Cx50 lines the pore of gap junction channels and plays an important role in single channel conductance and transjunctional voltage-dependent gating as well as in limiting the rate of ion permeation. The pore size of a gap junction channel, its switch control for opening and closing, and the modulations by chemicals can differ depending on the connexin subtypes that compose the channel. [21]. It is possible that in the heterozygous animals, heterotypic and/or heteromeric Cx50 channels are formed with both the mutated and the wild-type connexin50, resulting in distinctive shifts in functional properties of the connexin [22]. The ability of mutant Cx50 to oligomerize with wild type Cx50 and other connexins to form gap junction channels is well documented [23]; the ensuing functional consequences range from no effect to dominant negative inhibition of the channel function [23, 24]. While the observable effects of the complete mutation included relative protection of the SHR-*Dca*−/− rats from the diabetogenic effect of dexamethasone and decrease of SOD activity combined with increase of TBARS, the heterozygous animals displayed a phenotypic profile markedly distinct from both the parental SHR and the SHR-*Dca*−/− strains. It has been established that gap junctions-mediated signaling is critical for correct function of pancreatic β cells in response to glucose stimulation and pulsatile insulin release [25, 26]. Mice deficient in connexin36, the major connexin isoform in pancreatic β cells, are normoglycemic but are intolerant to postprandial glucose levels and show loss of circulating insulin oscillations, similar to human prediabetes [26, 27]. A recent study of 299 type 2 diabetics and 500 unrelated normoglycemic subjects corroborated these findings in human sample by showing that T allele of single nucleotide polymorphism rs3743123 in *GJD2* gene (coding for Cx36) leads to altered formation of gap junction plaques and cell coupling in β cells despite showing only marginal association to type 2 diabetes status [25]. There is further evidence showing that dexamethasone-induced insulin resistance is associated with increased connexin 36 mRNA and protein expression in pancreatic rat islets [28]. Although no data are so far available for the similar involvement of Cx50, it is of interest that according to data from the Human Integrated Protein Expression Database, CX50 protein is overexpressed in pancreas [29]. Nevertheless, this hypothesis remains to be validated by further studies. The significantly increased concentration of gastric inhibitory polypeptide together with highest insulinemia observed in heterozygotes is in line with the presumed primary action of GIP, i.e. the stimulation of glucose-dependent insulin secretion. Furthermore, the increase in postprandial insulin secretion after dexamethasone administration were shown to be mediated, at least in part, by increases in meal-stimulated GIP secretion [30]. One of the most striking observations was the simultaneous shift towards higher concentrations of triglycerides in chylomicrons and VLDL together with decrease of LDL and HDL cholesterol in heterozygous animals in comparison with both SHR and the SHR-*Dca*−/−. There is robust body of literature describing the role of connexins in mediating the unfavorable effects of hyperlipidemia [31–33], particularly the atherosclerotic plaque formation [34]. However, in the current study the cholesterol distribution into lipoprotein classes was modulated by distinct connexin 50 genotype, perhaps through non-genomic action of the glucocorticoid similar to the one described for Cx43 [35]. It is probable that the mutant Cx50 cannot form hemichannels or gap junctions in SHR-*Dca*−/− tissues [36] and their function related to lipid handling is compensated for by other members of the connexin family. However, in heterozygous animals, formation of heteromeric hemichannels containing both wild type and mutant Cx50 is possible with functional consequences (distinct permeability, voltage gating, ability to form homotypic or heterotypic gap junctions) leading, through yet unidentified mechanism, to the observed hyperlipidemia. Also, transdominant effects of the mutant Cx50 on other connexins cannot be ruled out as similar effects were described e.g. for interaction between mutant Cx26 and Cx43 [37]. Several of the increased oxidative stress indices were apparent in plasma and livers of rats with both homozygous and heterozygous form of the Cx50 mutation. Again, this trend was much more pronounced in SHR-*Dca*+/− rats, with exclusive manifestation also in kidney (decrease of activity glutathione peroxidase and superoxide dismutase). This was accompanied by increased levels of pro-inflammatory cytokines including IL-6, IL-12, IL-17 and TNF-α. The role of oxidative stress and inflammation in cardiovascular disease and related conditions is well established and is affected by numerous intrinsic and extrinsic factors [38]. Several connexins were previously linked to redox homeostasis, including Cx40 [39], Cx43 [40] and Cx46 [41]. The mechanism of their involvement include intra- and extracellular exchange of small molecules that are critical for redox homeostasis, such as GSH [42], for which Cx50 was shown to be permeable [41]. The heteromeric channels containing the mutant Cx50 could thus affect the redox homeostasis directly by modulating the flow of relevant ions, molecules or metabolites or through altered post-translational modifications and interactions with other cellular proteins [43]. One of the limitations of the present study lies in the fact that we did not uncover the detailed mechanism which underlies the metabolic effects in the *Cx50-*deficient and heterozygous animals. Also, since all the measurements were done in animals treated with dexamethasone, the specific outcomes may be due to pharmacogenetic interaction of variant Cx50 with dexamethasone as glucocorticoid effects on connexins have been documented [35].

## Conclusions

The results of this study demonstrate that homozygous mutation in the amino terminal domain of Cx50 leads, in dexamethasone-treated adult male rats, to relative protection from dexamethasone-induced insulin resistance, decrease in visceral adiposity and increase in oxidative stress indices. At the same time, heterozygous form of the same Cx50 variant results in increased concentration of triacylglycerols, decrease of cholesterol and elevation of several pro-inflammatory cytokines. Altogether, we show the substantial involvement of connexin 50 in several metabolic syndrome features.

## Additional file

Additional file 1:
**Table S1.** Lipoprotein particle size in SHR, SHR-*Dca*+/- and SHR-*Dca*-/- male rats. (PDF 114 kb)

## Abbreviations

C: Cholesterol
CAT: Catalase
CD: Conjugated dienes
CM: Chylomicron
Cx26: Connexin26
Cx36: Connexin36
Cx40: Connexin40
Cx43: Connexin43
Cx46: Connexin46
Cx50: Connexin50
EPF: Epididymal fat pad
EPO: Erythropoietin
G-CSF: Granulocyte-colony stimulating factor
GIP: Gastric inhibitory peptide
*Gja8* gene: Gap junction protein, alpha 8 coding for connexin50 gene
*GJD2* gene: Gap junction protein, delta 2 coding for connexin36 gene
GLP1: Glucagon-like polypeptide-1
GM-CSF: Granulocyte-macrophage colony-stimulating factor
GR: Glutathione reductase
GRO/KC: Chemokine (C-X-C motif) ligand 1
GSH: Glutathione
GSH-Px: Glutathione peroxidase
HDL: High-density lipoprotein
IFN-γ: Interferon gamma
IL-10: Interleukin 10
IL-12(p70): Interleukin 12(p70)
IL-13: Interleukin 13
IL-17A: Interleukin 17A
IL-18: Interleukin 18
IL-1α: Interleukin 1α
IL-1β: Interleukin 1β
IL-2: Interleukin 2
IL-4: Interleukin 4
IL-5: Interleukin 5
IL-6: Interleukin 6
IL-7: Interleukin 7
LDL: Low-density lipoprotein
MCP-1: Monocyte chemotactic protein 1
M-CSF: Macrophage colony-stimulating factor
MIP-1α: Macrophage inflammatory protein 1-alpha
MIP-3α: Macrophage inflammatory protein 3-alpha
OGTT: Oral glucose tolerance test
PP: Pancreatic polypeptide
PYY: Protein tyrosine tyrosine
RANTES: Regulated on activation, normal T cell expressed and secreted
RPF: Retroperitoneal fat pad
SHR: Spontaneously hypertensive rat
SHR-*Dca*−/−: Coisogenic SHR strain with a spontaneous mutation (homozygous) L7Q in *Gja8* gene, SHR-Gja8^m1Cub−/−^
SHR-*Dca*+/−: Coisogenic SHR strain with a spontaneous mutation (heterozygous) L7Q in *Gja8* gene, SHR-Gja8^m1Cub+/−^
SOD: Superoxide dismutase
TBARS: Thiobarbituric acid reactive substances
TG: Triacylglycerols
TNF-α: Tumor necrosis factor alpha
VEGF: Vascular endothelial growth factor
VLDL: Very low-density lipoprotein
