# Supplementary material for: Heterozygous connexin 50 mutation affects metabolic syndrome attributes in spontaneously hypertensive rat
Source: Lipids Health Dis. 2016 Nov 21;15:199. doi: 10.1186/s12944-016-0376-3 (PMC5117636; doi:10.1186/s12944-016-0376-3)
Supplement: Additional file 1: — Table S1. Lipoprotein particle size in SHR, SHR-Dca+/- and SHR-Dca-/- male rats. (PDF 114 kb) [file 12944_2016_376_MOESM1_ESM.pdf]

Supplementary Table 1. Lipoprotein particle size in SHR, SHR-*Dca*+/- and SHR-*Dca*-/- male rats.

**Trait (nm)**	**SHR**	**SHR-*Dca* +/-**	**SHR-*Dca* -/-**	**P_{ANOVA}**
VLDL	51.9 ± 0.3*	52.8 ± 0.3*, [a]	51.9 ± 0.2	**0.038**
LDL	19.3 ± 0.1	19.8 ± 0.3	19.5 ± 0.2	0.37
HDL	13.1 ± 0.02	12.8 ± 0.1	13.0 ± 0.09	0.16

Supplementary Table 1. Lipoprotein particle (VLDL...very low-density lipoprotein, LDL...low-density lipoprotein, HDL...high-density lipoprotein) size in SHR, SHR-*Dca*+/- and SHR-*Dca*-/- adult male rats. Data are shown as mean ± S.E.M. The significance levels of one-way ANOVA for STRAIN as a major factor are shown in last column. The significance levels for pair-wise, inter-strain comparisons between SHR-*Dca*-/- and SHR-*Dca*+/- strains vs. SHR are shown for post-hoc Tukey's HSD test as follows: *… P < 0.05; a… P < 0.05 for the differences between SHR-*Dca*-/- and SHR-*Dca*+/- strains.
